# Sublingual microcirculation-derived glycocalyx barrier impairment independently predicts progression of asymptomatic left ventricular diastolic dysfunction in elderly Chinese adults

**DOI:** 10.3389/fcvm.2026.1933360

**Published:** 2026-09-24

**Authors:** Xianxin Meng, Shaoyan Liu, Shen Liu, Meng Xie, Yang Liu

**Affiliations:** 1 Department of Cardiovascular Ultrasound, The Fourth People’s Hospital of Jinan, Jinan, China; 2 Department of Cardiology, Yantai Municipal Laiyang Central Hospital, Yantai, China; 3 The 13th Retired Cadres Rest Home of Haidian District, Beijing, China; 4 Medical School of Chinese People’s Liberation Army, Beijing, China; 5 Department of Cardiology, Second Medical Center of Chinese People’s Liberation Army General Hospital, Beijing, China; 6 National Clinical Research Center for Geriatric Diseases, Beijing, China; 7 Interventional Ultrasound Department, Beijing Anzhen Hospital Affiliated to Capital Medical University, Beijing, China

**Keywords:** endothelial glycocalyx, heart failure with preserved ejection fraction, left ventricular diastolic dysfunction, perfused boundary region, sublingual microcirculation

## Abstract

**Background:**

Coronary microvascular dysfunction has been confirmed to be associated with cardiac diastolic dysfunction (DD). The endothelial glycocalyx (EG) is essential for maintaining microvascular homeostasis, and its degradation is related to coronary microvascular dysfunction. However, direct assessment of coronary EG integrity remains technically challenging. Notably, the sublingual microcirculation provides a non-invasive window into systemic microvascular health, which is relevant to coronary microvascular status in conditions of diffuse endothelial dysfunction. Whether EG integrity assessed at the sublingual site is associated with the presence and progression of DD remains unclear.

**Methods:**

This prospective observational study enrolled 503 retired workers aged ≥60 years without overt heart failure from a single center in China. EG integrity was assessed at baseline using sidestream dark field imaging-derived perfused boundary region (PBR) of the sublingual microcirculation, with a higher PBR indicating greater glycocalyx barrier impairment. Diastolic function was evaluated by echocardiography according to the 2016 ASE/EACVI criteria at baseline and after a median follow-up of 23 months. Multivariable logistic regression and linear mixed-effects models were used to evaluate cross-sectional and longitudinal associations.

**Results:**

The prevalence of ALVDD at baseline was 9.74% (*n* = 49). PBR was significantly higher in participants with ALVDD than in those with normal diastolic function (2.43 vs. 1.84 μm, *P* < 0.01). After adjustment for the HOMAGE score, each 0.1 μm increase in PBR was independently associated with a 12% higher odds of ALVDD (OR 1.12, 95% CI 1.06-1.18, *P* < 0.01). Adding PBR to the HOMAGE score significantly improved discriminative ability (AUC from 0.656 to 0.712, *P* = 0.04; NRI 0.73, *P* = 0.02). During follow up, participants with higher baseline PBR showed significantly greater annual deteriorations in E/e’ ratio and septal/lateral e' velocities compared with those with lower PBR (all *P* for trend <0.01).

**Conclusions:**

Greater EG barrier impairment, non-invasively quantified by sublingual PBR, is independently associated with both the presence and longitudinal progression of ALVDD in an elderly Chinese population. PBR may serve as a useful marker for early detection of diastolic deterioration and and raises the hypothesis that PBR could serve as a potential target for preventive interventions, which requires prospective validation.

## Introduction

Heart failure with preserved ejection fraction (HFpEF) is a multifaceted clinical entity associated with substantial morbidity and mortality (1, 2). Prior to the overt manifestation of HFpEF, a clinically silent stage exists, referred to as asymptomatic left ventricular diastolic dysfunction (ALVDD). Given the limited therapeutic options for reversing established HFpEF, interventions targeting the prevention of ALVDD development are considered a strategic priority (3). Consequently, identifying factors associated with ALVDD onset offers a promising avenue for addressing current therapeutic challenges in HFpEF.

Accumulating evidence indicates that endothelial inflammation is a key contributor to left ventricular diastolic dysfunction (LVDD) (4–6). Inflammatory processes within the endothelium reduce nitric oxide (NO) availability in adjacent cardiomyocytes by impairing endothelium-derived NO signaling. Through the NO-soluble guanylate cyclase (sGC)-cyclic guanosine monophosphate (cGMP)-protein kinase G (PKG) signaling cascade, diminished NO bioavailability results in reduced PKG activity. This, in turn, promotes cardiomyocyte hypertrophy, increases myocyte stiffness, and facilitates interstitial collagen accumulation—all hallmarks of LVDD (7).

Multiple factors modulate the intensity of endothelial inflammation. Among them, the endothelial glycocalyx (EG) lining the vascular lumen plays a crucial role. The EG is essential for preserving microvascular homeostasis through regulation of vascular resistance, signal transduction, and protection against circulating inflammatory mediators and cells, all of which modulate the severity of endothelial inflammation. Structurally, the EG is a gel-like, carbohydrate-rich layer coating the endothelial surface, composed of glycosaminoglycans, glycoproteins, glycolipids, and proteoglycans, including the syndecan family (8, 9). Direct visualization of the EG *in vivo* remains technically challenging; however, EG integrity can be assessed indirectly. Of note, sublingual and coronary microvessels share embryonic homology, both originating from the mesoderm and belonging to the terminal microvascular beds of the systemic circulation. Thus, the sublingual microcirculation provides a non-invasive window into systemic microvascular integrity, which is known to mirror, at least in part, coronary microvascular status in conditions characterized by diffuse endothelial dysfunction (10, 11). A well-validated non-invasive approach is sidestream dark field (SDF) imaging of the sublingual microcirculation, which yields a dynamic measure termed the perfused boundary region (PBR). An enlarged PBR reflects a thinner or damaged EG, as deeper penetration of red blood cells into the glycocalyx layer indicates impaired barrier function (12). Previous studies have provided supportive evidence linking PBR to coronary microvascular status (13). Elevated PBR has been independently associated with reduced left ventricular systolic function and adverse remodeling after acute myocardial infarction (14). These findings reinforce the notion that PBR is not merely a local glycocalyx measure but also reflects the status of the coronary microvascular bed.

Building upon the recognized contribution of the EG to endothelial inflammation and the established link between coronary endothelial inflammation and LVDD, we hypothesized that EG status is a significant determinant of LVDD occurrence. Therefore, the present study aimed to investigate the relationship between EG integrity and ALVDD, employing SDF imaging-derived PBR of sublingual microvessels as a non-invasive assessment. Furthermore, we correlated these PBR measurements with echocardiographic parameters of diastolic function.

## Methods

### Study design and subjects

This was a single-centre, prospective observational study conducted at Beijing Anzhen Hospital, China. Consecutive retired workers aged ≥60 years who underwent routine annual physical examinations between January and December 2023 were screened for eligibility. Exclusion criteria were: (i) history of malignant tumours or rheumatic immune diseases; (ii) previous coronary intervention, documented myocardial infarction, or electrocardiographic/ echocardiographic signs of prior infarction (pathological Q waves or regional wall motion abnormalities); (iii) cardiomyopathy, severe valvular heart disease, myocarditis, or symptomatic congestive heart failure; and (iv) major surgery or severe infection within the preceding three months. The study protocol was approved by the Ethics Committee of Beijing Anzhen Hospital (KS2022057), and all participants provided written informed consent.

All enrolled individuals underwent a comprehensive baseline evaluation, including anthropometric measurements, fasting blood sampling (routine haematology, biochemistry, and NT-proBNP), echocardiography, and non-invasive assessment of EG integrity. After a median follow-up of 23 months (re-examination in 2025), a second echocardiographic examination was performed to assess longitudinal changes in diastolic function. Laboratory tests were not repeated at the follow-up visit.

### Non-invasive assessment of EG integrity

EG status was evaluated by measuring the PBR of the sublingual microcirculation using SDF imaging (V210, Microsense, MedSoft System, Guangzhou, China). The technique has been validated in previous studies (12, 15).

The imaging device uses a green light-emitting diode (540 nm), and the light is reflected by haemoglobin in red blood cells (RBCs), enabling visualization of RBC movement within microvessels. The disposable probe was placed gently under the tongue, and the system automatically recorded approximately 3000 vascular segments with diameters ranging from 4 to 25μm. Recordings with contrast enhancement below 60% were automatically rejected. Beyond contrast enhancement, the software applies additional automated quality control criteria to ensure measurement reliability. Recordings are assessed for brightness, focus, image stability, and freedom from pressure artefacts, consistent with established quality scoring systems for SDF imaging (16). For each recorded vessel segment, the software verifies that red blood cell filling is ≥50%, red blood cell column width is ≥2 μm, and that the segment is not curved or overlapping with adjacent segments. The system continues data acquisition until approximately 3,000 valid vessel segments are captured. These criteria ensure that only high-quality vessel segments are included in the final PBR calculation, minimising measurement bias.

For each valid vessel segment, the software determined the total perfused diameter and the width of the RBC column. PBR was calculated as:

### PBR (μm) = (perfused diameter—RBC column width)/2

A larger PBR indicates deeper penetration of RBCs into the glycocalyx layer, reflecting reduced glycocalyx thickness and impaired barrier integrity, whereas a smaller PBR corresponds to a robust, intact glycocalyx. The software provides mean PBR values for vessels of 4-9μm, 10-19μm, and 20-25μm, as well as the overall average for 4-25μm. In this study, we used the overall PBR as the primary indicator of glycocalyx integrity. The measurement is operator-independent, requires approximately 3 min, and demonstrates good reproducibility (reported inter-observer variability of 5.1% and intra-observer variability of 4.9%) (15). Because the software only analyzes vessel segments with a filling percentage >50% and at least 11 of 21 line markers detecting RBCs, PBR values are not influenced by haematocrit variations. To assess reproducibility, 30 randomly selected participants underwent two sublingual imaging sessions 2 h apart, performed by two independent operators (the second blinded to the first). Inter- and intra-observer variability were assessed by calculating the coefficient of variation (CoV), with a CoV <10% considered acceptable.

### Echocardiography and assessment of diastolic function

Echocardiographic examinations were performed using a GE Vivid 7 ultrasound system equipped with a 2.5 MHz M3S probe. All measurements followed the 2016 recommendations of the American Society of Echocardiography and the European Association of Cardiovascular Imaging (17). Standard two-dimensional and M-mode images were obtained from parasternal long-axis, short-axis, apical two-chamber, and four-chamber views.

Left ventricular ejection fraction (LVEF) was calculated using the biplane Simpson's method. Left ventricular mass was derived using the Devereux formula and indexed to body surface area (LVMI). Left atrial volume was measured using the biplane modified Simpson's method at end-systole and indexed to body surface area (LAVI).

Diastolic function was evaluated using pulsed-wave Doppler and tissue Doppler imaging. The following parameters were recorded: early (E) and late (A) mitral inflow velocities, E/A ratio, septal and lateral early diastolic annular velocities (e'), average E/e' ratio, tricuspid regurgitation (TR) velocity, and LAVI. According to the 2016 ASE/EACVI algorithm, diastolic dysfunction (DD) was diagnosed when more than half of the four criteria were positive: average E/e' > 14, septal e' < 7 cm/s or lateral e' < 10 cm/s, TR velocity >2.8 m/s, and LAVI >34 mL/m^2^. Participants with fewer than 50% positive parameters were classified as having normal diastolic function (CON), those with exactly 50% as indeterminate (INDER), and those with more than 50% as having DD.

### Laboratory measurements

Fasting venous blood samples were collected on the morning of the physical examination. Routine biochemical indices (fasting glucose, creatinine, total cholesterol, triglycerides, LDL-cholesterol, HDL-cholesterol, AST, and ALT) were measured using an automatic analyzer (7600P, HITACHI, Tokyo, Japan). NT-proBNP was determined by electrochemiluminescence immunoassay (Roche Diagnostics GmbH). The HOMAGE score, a validated composite risk score for heart failure, was calculated for each participant using the published formula incorporating sex, age, body mass index, smoking status, history of diabetes or coronary artery disease, use of antihypertensive medication, systolic blood pressure, heart rate, and creatinine (18).

### Statistical analysis

Continuous variables are presented as mea*n* ± standard deviation (SD) if normally distributed, or as median with interquartile range (IQR) if non-normally distributed. Categorical variables are expressed as frequencies and percentages. Comparisons between groups were performed using Student's t-test, Mann–Whitney U test, or *χ*^2^ test, as appropriate. For comparisons involving more than two groups, the Kruskal–Wallis test was used for non-normally distributed continuous variables, followed by Dunn's *post-hoc* test with appropriate correction for multiple comparisons when the overall test was significant.

The median PBR value of the whole cohort was used as an exploratory cut-off to define a high PBR group (HPG) and a low PBR group (LPG). Baseline characteristics were compared between these two groups. Subsequently, participants were categorized into CON, INDER, and DD groups according to the 2016 diastolic function guidelines, and baseline data were compared across the three groups.

Multivariable logistic regression was used to assess factors independently associated with DD. Receiver operating characteristic (ROC) curves were constructed to evaluate the ability of PBR, HOMAGE score, and their combination to discriminate DD. The area under the curve (AUC) was calculated and comparison between AUCs was performed using DeLong's test, and the optimal cut-off value for PBR was determined using the Youden index. The discriminative performance of PBR was further evaluated using net reclassification improvement (NRI) and integrated discrimination improvement (IDI).

To explore whether the PBR-ALVDD association varied across key subgroups, we performed pre-specified subgroup analyses stratified by sex, age (<65 vs. ≥ 65 years), diabetes, hypertension, and smoking status. For each subgroup, we ran separate multivariable logistic regression models adjusted for the HOMAGE score (excluding the stratification variable itself to avoid collinearity). Heterogeneity was tested by introducing multiplicative interaction terms (PBR ×  subgroup) into the full model; a *P* for interaction <0.10 was considered suggestive of effect modification.

To evaluate sample size adequacy, we calculated the events-per-variable (EPV) ratio. The primary model (HOMAGE + PBR) had an EPV of 24.5 (49 events/2 variables), exceeding the recommended minimum of 10. *Post-hoc* power analysis indicated that with 49 events, the study had >80% power to detect the observed OR of 1.12. The extended sensitivity model (7 covariates) had an EPV of 7; we therefore interpret it as confirmatory rather than definitive.

For longitudinal analysis, linear mixed-effects models were applied to estimate annual changes in echocardiographic parameters (E/e', septal e', lateral e', LAVI, and TR velocity). The models were adjusted for baseline age, sex, BMI, diabetes, CKD, and the baseline value of the respective echocardiographic parameter to account for regression to the mean. The interaction term between time and PBR group (HPG vs. LPG) was used to test whether the rate of change differed significantly between groups; the *P*-value for this interaction is reported as P_trend_. To assess attrition bias, we compared baseline characteristics between participants who completed follow-up and those lost to follow-up (Table 4). No significant differences were observed for any key variable (all *P* > 0.05). Given the low (8.5%) and balanced attrition, no additional imputation was performed. A two-sided *P*-value < 0.05 was considered statistically significant. All statistical analyses and graphs were generated using R software version 4.5.2 (R Foundation for Statistical Computing, Vienna, Austria).

### AI-assisted language tools

During the preparation of this manuscript, the authors used AI-assisted language polishing tools (specifically, Claude 3.5 Sonnet and Grammarly) solely for grammar checking, language refinement, and clarity improvement. No AI tool was used for data analysis, statistical computation, result interpretation, or generation of scientific content. All data analyses were performed using R software version 4.5.2, and all scientific conclusions were drawn by the authors. The authors take full responsibility for the integrity and accuracy of the data and the final manuscript.

## Results

### Baseline characteristic of enrolled patients

A total of 503 elderly individuals (mean age 66 ± 5 years, 81.51% male) were enrolled. The median PBR in the whole cohort was 2.08 μm [interquartile range (IQR): 1.76-2.47 μm]. Reproducibility analysis in our cohort (*n* = 30 randomly selected participants) demonstrated excellent agreement, with an inter-observer coefficient of variation of 5.2% (95% CI: 4.1%—6.3%) and an intra-observer coefficient of variation of 4.6% (95% CI: 3.6%-5.6%). These values are comparable to those reported in previous methodological studies (15) and confirm that PBR measurements in our hands are highly reproducible. Since no universally accepted clinical cut-off exists for PBR, we used the cohort median as an exploratory threshold to define a high PBR group (HPG: PBR ≥ 2.08μm, *n* = 252) and a low PBR group (LPG: PBR < 2.08 μm, *n* = 251). This allowed comparison of baseline characteristics between individuals with relatively greater versus lesser glycocalyx barrier impairment. A more optimal cut-off for discriminating diastolic dysfunction was subsequently derived from ROC analysis.

Table 1 summarizes the baseline demographic, clinical, laboratory, and echocardiographic parameters according to PBR group. Compared with the LPG, patients in the HPG were older (68 ± 7 vs. 64 ± 4 years, *P* = 0.01) and less frequently male (68.25% vs. 94.82%, *P* < 0.01). The prevalence of diabetes mellitus (19.44% vs. 12.35%, *P* = 0.02), chronic kidney disease (12.70% vs. 6.77%, *P* = 0.02) and current smoker (42.86% vs. 23.11%, *P* < 0.01) was significantly higher in the HPG. Body mass index was also significantly higher in the HPG (median 27.2 vs. 25.9 kg/m^2^, *P* = 0.03). The median systolic blood pressure was higher in the HPG than in the LPG (141 vs. 135 mmHg, *P* = 0.02), while diastolic blood pressure was slightly lower in the HPG (71 vs. 73 mmHg, *P* = 0.04), consistent with the lower proportion of males in this group. Other cardiovascular risk factors and lipid profiles did not differ significantly between the two groups. As shown in Table 1, the proportion of DD was markedly higher in the HPG than in the LPG (15.87% vs. 3.58%, *P* < 0.01), while the proportion of normal function was lower (67.86% vs. 80.88%, *P* < 0.01).

**Table 1:** Baseline characteristics and echocardiographic parameters of participants stratified by the median perfused boundary region.

Characteristic	All (*n* = 503)	HPG (*n* = 252)	LPG (*n* = 251)	*P* value
Demographic data				
Age, years	66 ± 5	68 ± 7	64 ± 4	0.01
Male, *n* (%)	410 (81.51)	172 (68.25)	238 (94.82)	<0.01
Body mass index, kg/m^2^	26.5 (24.1–29.3)	27.2 (24.8–30.1)	25.9 (23.5–28.6)	0.03
Current smoker, *n* (%)	166 (33.00)	108 (42.86%)	58 (23.11)	<0.01
Comorbidities, *n* (%)				
Diabetes mellitus	80 (15.90)	49 (19.44)	31 (12.35)	0.02
Hypertension	208 (41.35)	108 (42.86)	100 (39.84)	0.42
Systolic BP, mmHg	138 (127–149)	141 (130–152)	135 (124–146)	0.02
Diastolic BP, mmHg	72 (65–78)	71 (64–77)	73 (66–79)	0.04
Antihypertensive use, *n* (%)	158 (31.4)	81 (32.1)	77 (30.7)	0.73
Chronic kidney disease	49 (9.74)	32 (12.70)	17 (6.77)	0.02
Laboratory parameters				
Fasting glucose, mmol/L	5.6 (5.1–6.4)	5.7 (5.2–6.6)	5.5 (5.0–6.2)	0.09
Total cholesterol, mmol/L	4.5 (3.9–5.2)	4.6 (4.0–5.3)	4.4 (3.8–5.1)	0.12
LDL cholesterol, mmol/L	2.7 (2.2–3.3)	2.8 (2.3–3.4)	2.6 (2.1–3.2)	0.08
HDL cholesterol, mmol/L	1.2 (1.0–1.4)	1.1 (0.9–1.3)	1.2 (1.0–1.3)	0.87
Triglycerides, mmol/L	1.5 (1.1–2.1)	1.6 (1.2–2.2)	1.4 (1.0–2.0)	0.06
Creatinine, μmol/L	71 (62–82)	73 (64–85)	69 (60–79)	0.03
eGFR, mL/min/1.73 m^2^	84 (72–96)	81 (68–92)	87 (75–99)	0.02
NT-proBNP, pg/mL	68 (52–95)	69 (53–98)	67 (50–92)	0.35
PBR measurement				
PBR, μm	2.08 (1.76–2.47)	2.31 (2.18–2.49)	1.89 (1.74–2.00)	<0.01
Echocardiographic parameters				
LVEF, %	62 (58–66)	61 (57–65)	63 (59–67)	0.07
LV mass index, g/m^2^	84 (75–104)	90 (79–105)	79 (65–100)	0.03
LAVI, mL/m^2^	33 (30–39)	36 (32–40)	32 (30–36)	0.18
E/A ratio	0.96 (0.83–1.11)	0.95 (0.80–1.12)	0.96 (0.85–1.11)	0.42
Average E/e'	11.12 (10.15–12.23)	10.87 (9.57–12.18)	11.32 (10.28–12.24)	0.07
Septal e', cm/s	8.19 (7.11–9.20)	7.75 (6.68–9.02)	8.49 (7.46–9.44)	<0.01
Lateral e', cm/s	9.81 (8.77–10.41)	9.60 (8.11–10.29)	9.95 (9.08–10.47)	<0.01
TR velocity, m/s	2.55 (2.47–2.63)	2.58 (2.49–2.68)	2.52 (2.44–2.60)	0.02
Diastolic dysfunction status, *n* (%)				
CON	374 (74.35)	171 (67.86)	203 (80.88)	<0.01
INDER	80 (15.90)	41 (16.27)	39 (15.54)	0.69
DD	49 (9.74)	40 (15.87)	9 (3.58)	<0.01
HOMAGE score	9.85 (7.92–13.04)	9.88 (7.92–13.01)	9.82 (8.09–13.06)	0.94

Data are presented as mean ± SD, median (IQR), or *n* (%). *P* values were calculated using Student's t-test, Mann–Whitney U test, or *χ*^2^ test, as appropriate. HPG, high PBR group; LPG, low PBR group; PBR, perfused boundary region; LDL, low-density lipoprotein; HDL, high-density lipoprotein; eGFR, estimated glomerular filtration rate; NT-proBNP, N-terminal pro-B-type natriuretic peptide; LVEF, left ventricular ejection fraction; LAVI, left atrial volume index; TR, tricuspid regurgitation; CON, normal diastolic function; INDER, indeterminate diastolic function; DD, diastolic dysfunction; HOMAGE, Heart ‘OMics’ in AGEing.

When participants were classified by diastolic function status according to the 2016 ASE/EACVI guidelines, 49 (9.74%) had definite diastolic dysfunction (DD), 80 (15.90%) had indeterminate function (INDER), and 374 (74.35%) had normal diastolic function (CON). The relevant data are presented in Table 2. The mean age of the LVDD group was significantly higher than that of both the INDER and CON groups (71 ± 7 vs. 67 ± 6 vs. 64 ± 5 years, *P* < 0.05 for both comparisons.). A stepwise trend was observed for sex distribution, body mass index, and the prevalence of diabetes mellitus and chronic kidney disease: the LVDD group had the highest BMI (median 27.8 kg/m^2^), highest prevalence of diabetes (22.4%) and CKD (14.3%), and the lowest proportion of males (65.3%), followed by the INDER group, whereas the CON group showed the most favorable profile (all *P* < 0.05 for trend). Median systolic blood pressure increased stepwise across the CON, INDER, and LVDD groups(135, 140, and 146 mmHg, *P* = 0.01) as did median diastolic blood pressure (71, 72, and 74 mmHg, *P* = 0.03) was observed across CON, INDER, and LVDD groups. Antihypertensive medication use tended to be higher in the LVDD group (42.9%) than in the CON (29.7%) and INDER groups (32.5%), though this did not reach statistical significance (*P* = 0.06). No significant differences were found in laboratory parameters (fasting glucose, lipid profile) or echocardiographic indices of systolic function and chamber dimensions among the three groups, except for eGFR, which decreased stepwise (87, 83, 80 mL/min/1.73 m^2^, *P* = 0.01).

**Table 2:** Baseline characteristics of participants according to diastolic function status.

Characteristic	CON (*n* = 374)	INDER (*n* = 80)	LVDD (*n* = 49)	*P* value
Demographic data				
Age, years	64 ± 5	67 ± 6	71 ± 7	0.03
Male, *n* (%)	318 (85.0)	60 (75.0)	32 (65.3)	<0.01
Body mass index, kg/m^2^	25.9 (23.5–28.6)	26.8 (24.5–29.2)	27.8 (25.0–30.5)	0.03
Current smoker, *n* (%)	124 (33.2)	26 (32.5)	16 (32.7)	0.96
Comorbidities, *n* (%)				
Diabetes mellitus	52 (13.9%)	18 (22.5%)	10 (20.4%)	0.02
Hypertension	150 (40.1)	35 (43.8)	23 (46.9)	0.18
Systolic BP, mmHg	135 (124–146)	140 (130–152)	146 (134–158)	0.01
Diastolic BP, mmHg	71 (64–77)	72 (65–78)	74 (67–80)	0.03
Antihypertensive use, *n* (%)	111 (29.7)	26 (32.5)	21 (42.9)	0.06
Chronic kidney disease	28 (7.5%)	13 (16.3%)	8 (16.3%)	0.02
**Laboratory parameters**				
Fasting glucose, mmol/L	5.5 (5.0–6.2)	5.6 (5.1–6.4)	5.7 (5.2–6.6)	0.12
Total cholesterol, mmol/L	4.4 (3.8–5.1)	4.5 (3.9–5.2)	4.6 (4.0–5.3)	0.22
LDL cholesterol, mmol/L	2.6 (2.1–3.2)	2.7 (2.2–3.3)	2.8 (2.3–3.4)	0.09
HDL cholesterol, mmol/L	1.2 (1.0–1.5)	1.0 (0.8–1.2)	1.1 (0.9–1.3)	0.16
Triglycerides, mmol/L	1.4 (1.0–2.0)	1.5 (1.1–2.1)	1.6 (1.2–2.2)	0.09
eGFR, mL/min/1.73 m^2^	87 (75–99)	83 (70–95)	80 (67–91)	0.01
NT-proBNP, pg/mL	66 (54–92)	68 (56–95)	71 (58–98)	0.28
**PBR measurement**				
PBR, μm	1.84 (1.62–2.09)	1.96 (1.71–2.23)	2.43 (2.08–2.82)	<0.01
**Echocardiographic parameters**				
LVEF, %	63 (59–67)	62 (58–66)	61 (57–65)	0.07
LV mass index, g/m^2^	84 (74–94)	88 (77–98)	92 (79–105)	0.01
LAVI, mL/m^2^	31 (28–35)	35 (31–39)	38 (34–42)	<0.01
E/A ratio	0.98 (0.86–1.12)	0.95 (0.82–1.09)	0.90 (0.76–1.04)	0.08
Average E/e'	10.5 (9.4–11.6)	12.0 (10.8–13.2)	14.1 (12.5–15.8)	<0.01
Septal e', cm/s	8.8 (7.9–9.7)	7.5 (6.6–8.4)	6.2 (5.4–7.0)	<0.01
Lateral e', cm/s	10.4 (9.5–11.3)	9.2 (8.3–10.1)	7.8 (6.9–8.7)	<0.01
TR velocity, m/s	2.48 (2.38–2.58)	2.61 (2.51–2.71)	2.79 (2.69–2.89)	<0.01
**HOMAGE score**	9.45 (7.8–12.6)	11.69 (8.77–13.75)	13.71 (11.8–15.32)	<0.01

Data are presented as mean ± SD, median (interquartile range), or number (%). CON, normal diastolic function; INDER, indeterminate diastolic function; LVDD, left ventricular diastolic dysfunction; other abbreviations as in Table 1.

The HOMAGE score, a validated composite predictor of heart failure risk, increased stepwise from CON to INDER to LVDD (9.45, 11.69, and 13.71, respectively, *P* < 0.01). No significant difference was observed between the HPG and LPG [9.88 (7.92-13.01) vs. 9.82 (8.09-13.06), *P* = 0.94].

### Association between EG barrier impairment and cardiac diastolic function

The median PBR in this cohort was 2.08 μm (IQR: 1.76-2.47 μm). Significant differences in PBR were observed between the LVDD and CON groups (CON vs. LVDD: 1.84 [1.62-2.09] vs. 2.43 [2.08-2.82] μm, *P* < 0.01).

PBR values were further compared after stratifying patients according to the four major echocardiographic parameters used for LVDD assessment (E/e' > 14, septal e' < 7 cm/s or lateral e' < 10 cm/s, LAVI >34 mL/m^2^, and TR velocity >2.8 m/s). Significant differences were found between those with E/e' > 14 versus ≤14 (2.41 [2.11-2.88] vs. 1.96 [1.66-2.24] μm, *P* < 0.01); between those with septal e' < 7 or lateral e' < 10 versus those with septal e' ≥ 7 and lateral e' ≥ 10 (2.27 [1.97-2.64] vs. 1.92 [1.67-2.19]μm, *P* < 0.01); and between participants with LAVI >34 versus ≤34 mL/m^2^ (2.25 [1.98-2.57] vs. 2.04 [1.75-2.42] μm, *P* = 0.04). In contrast, no significant PBR difference was noted when comparing TR >2.8 m/s versus ≤2.8 m/s (2.12 [1.85-2.45] vs. 2.16 [1.84-2.53]-μm, *P* = 0.056). Figure 1 illustrates the PBR distribution across these subgroups.

**Figure 1:**
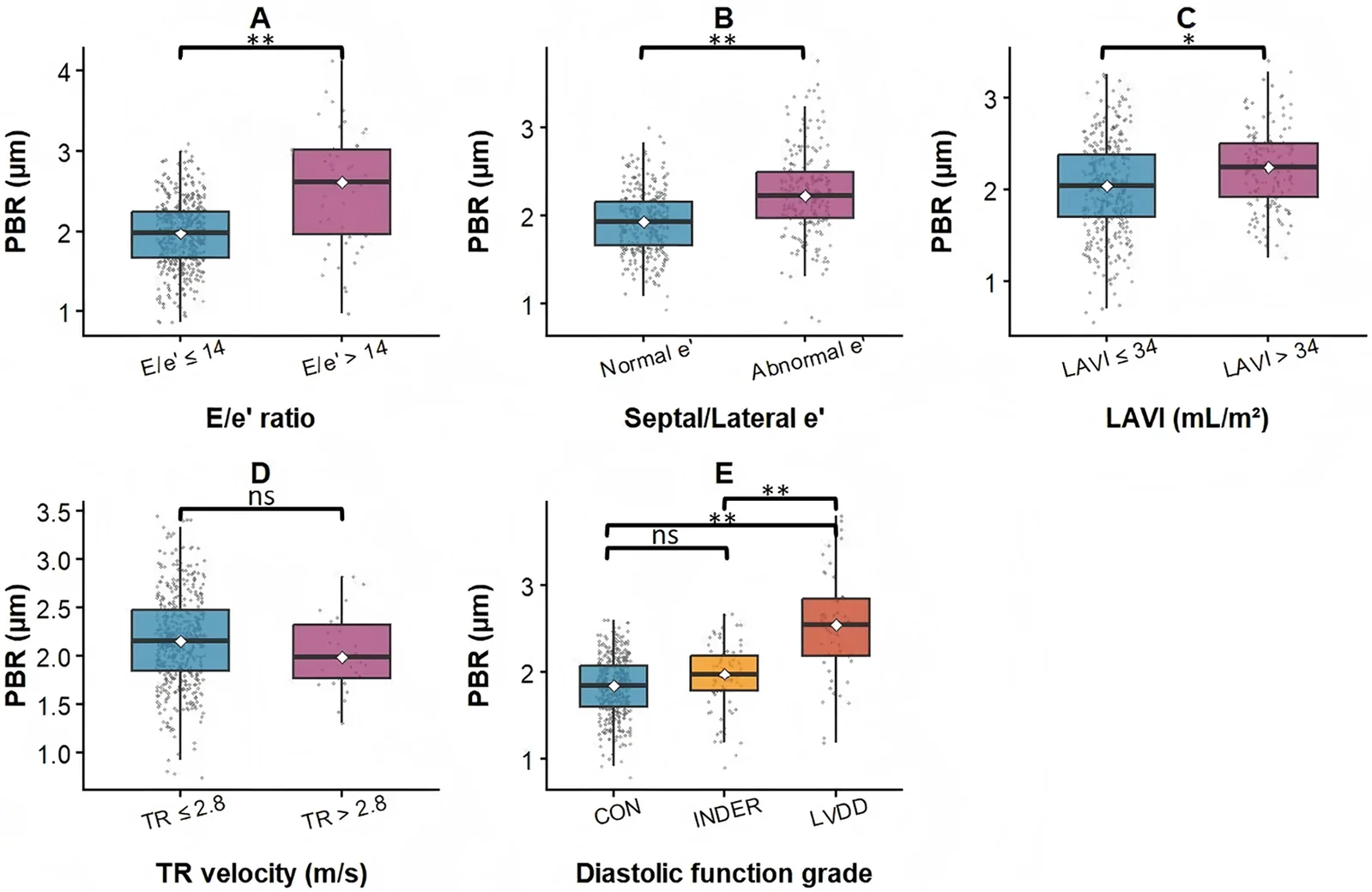
Box plots with overlaid individual data points showing the distribution of PBR values stratified by echocardiographic parameters and diastolic function grades. **(A)** Average E/e' ratio (≤14 vs. > 14); **(B)** septal e' (≥7 cm/s) and lateral e' (≥10 cm/s) vs. either septal e' < 7 cm/s or lateral e' < 10 cm/s; **(C)** LAVI (≤34 vs. > 34 mL/m^2^); **(D)** TR velocity (≤2.8 vs. > 2.8 m/s); **(E)** PBR distribution across diastolic function grades (CON, INDER, and LVDD). Boxes represent the IQR, horizontal lines indicate medians, whiskers extend to the minimum and maximum values (excluding outliers), and circles denote individual data points. Comparisons between groups were performed using the Mann–Whitney U test for panels A-D and the Kruskal–Wallis test with Dunn's *post-hoc* test for panel E. **P < 0.01, *P < 0.05; ns, not significant. CON, normal diastolic function; DD, diastolic dysfunction; INDER, indeterminate diastolic function; IQR, interquartile range; LAVI, left atrial volume index; PBR, perfused boundary region; TR, tricuspid regurgitation.

Given the limited number of DD events (*n* = 49) and to avoid overfitting, a parsimonious multivariable logistic regression model was prioritized. The primary model included HOMAGE score and PBR as the main predictors. Because the HOMAGE score already integrates age, sex, BMI, smoking status, diabetes, and renal function, entering these individual covariates simultaneously wit h the HOMAGE score would introduce collinearity. Therefore, individual covariates were not included in the primary model. Univariate analysis was first performed to identify factors associated with LVDD (Table 3). In the primary multivariable model (HOMAGE + PBR), PBR remained independently associated with LVDD (OR per 0.1-μm increase: 1.12, 95% CI: 1.06-1.18, *P* < 0.01). A sensitivity analysis additionally adjusting for age, sex, BMI, diabetes mellitus, chronic kidney disease, and hypertension (Model 2) confirmed the robustness of the main findings, with PBR remaining independently associated with LVDD (OR per 0.1-μm increase: 1.09, 95% CI: 1.04-1.15, *P* < 0.01; Table 3). Notably, although the extended sensitivity model incorporated more covariates at the cost of a lower EPV (7 vs. 24.5 in the primary model), the consistent OR estimates between the two models support the robustness of the primary finding and suggest that residual confounding from these covariates is unlikely to fully account for the observed association.

**Table 3:** Univariate and multivariable logistic regression analyses of factors associated with left ventricular diastolic dysfunction.

Factors	Univariate analysis		Model 1		Model 2	
	OR (95% CI)	*P*	OR (95% CI)	*P*	OR (95% CI)	*P*
Age, per year	1.12 (1.03–1.21)	0.01	-	-	1.09 (1.03–1.15)	0.03
Male sex	0.48 (0.25–0.92)	0.03	-	-	0.52 (0.23–1.12)	0.09
Body mass index, per kg/m^2^	1.18 (1.07–1.31)	0.01	-	-	1.07 (0.95–1.19)	0.20
Diabetes mellitus	2.51 (1.20–5.23)	0.01	-	-	2.10 (0.85–5.10)	0.10
Chronic kidney disease	2.65 (1.23–5.71)	0.01	-	-	1.75 (0.78–3.90)	0.16
Hypertension	1.98 (1.05–3.73)	0.03			1.57 (0.80–3.08)	0.18
HOMAGE score, per 1 unit	1.45 (1.28–1.63)	<0.01	1.40 (1.23–1.59)	<0.01	1.20 (1.01–1.42)	0.03
PBR, per 0.1 μm increase	1.12 (1.07–1.17)	<0.01	1.12 (1.06–1.18)	<0.01	1.09 (1.04–1.15)	<0.01

Data are presented as odds ratios (OR) with 95% confidence intervals (CI). Model 1: adjusted for HOMAGE score and PBR. Model 2: adjusted for HOMAGE score, age, sex, body mass index, diabetes mellitus, chronic kidney disease, and hypertension (defined as SBP ≥140 mmHg, DBP ≥90 mmHg, or use of antihypertensive medication). HOMAGE, Heart ‘OMics’ in AGEing; PBR, perfused boundary region.

### Subgroup analyses

To evaluate the consistency of the association between PBR and ALVDD across key clinical subgroups, we performed stratified analyses by sex, age (<65 vs. ≥ 65 years), diabetes mellitus, and hypertension, with formal tests for interaction (Supplementary Table S1). The adjusted odds ratios for ALVDD per 0.1μm increase in PBR were directionally consistent across all strata: 1.11 (95% CI: 1.05-1.17) in males and 1.14 (95% CI: 1.05-1.23) in females; 1.12 (95% CI: 1.04-1.20) in participants aged <65 years and 1.11 (95% CI: 1.05-1.18) in those aged ≥65 years; 1.10 (95% CI: 1.01-1.19) in participants with diabetes and 1.12 (95% CI: 1.06-1.19) in those without; and 1.11 (95% CI: 1.04-1.18) in hypertensive participants and 1.13 (95% CI: 1.06-1.20) in normotensive participants. None of the interaction terms reached statistical significance (all P for interaction >0.30), indicating that the PBR-ALVDD association was robust and not materially modified by these clinical characteristics.

### Discriminative value of EG barrier impairment for the presence of LVDD

Receiver operating characteristic (ROC) analysis was employed to evaluate the ability of PBR to discriminate LVDD (Figure 2). The area under the curve (AUC) for PBR was 0.564 [standard error (SE): 0.032, 95% CI: 0.471-0.597, *P* < 0.01]. For comparison, the HOMAGE score yielded an AUC of 0.656 (SE: 0.041, 95% CI: 0.576-0.735, *P* < 0.01). When PBR and HOMAGE score were combined, the AUC increased to 0.712 (SE: 0.036, 95% CI: 0.642-0.782, *P* < 0.01). DeLong's test confirmed a statistically significant improvement in AUC when PBR was added to the HOMAGE score (*P* = 0.04). The exploratory optimal cut-off values derived from ROC analysis in this cohort were 2.29μm for PBR and 11.65 for the HOMAGE score, providing sensitivities/specificities of 63.5%/53.4% for PBR and 68.6%/59.6% for HOMAGE score. These cut-offs are study-specific and require external validation before clinical application. The combined model achieved a sensitivity of 70.5% and a specificity of 69.8%.

**Figure 2:**
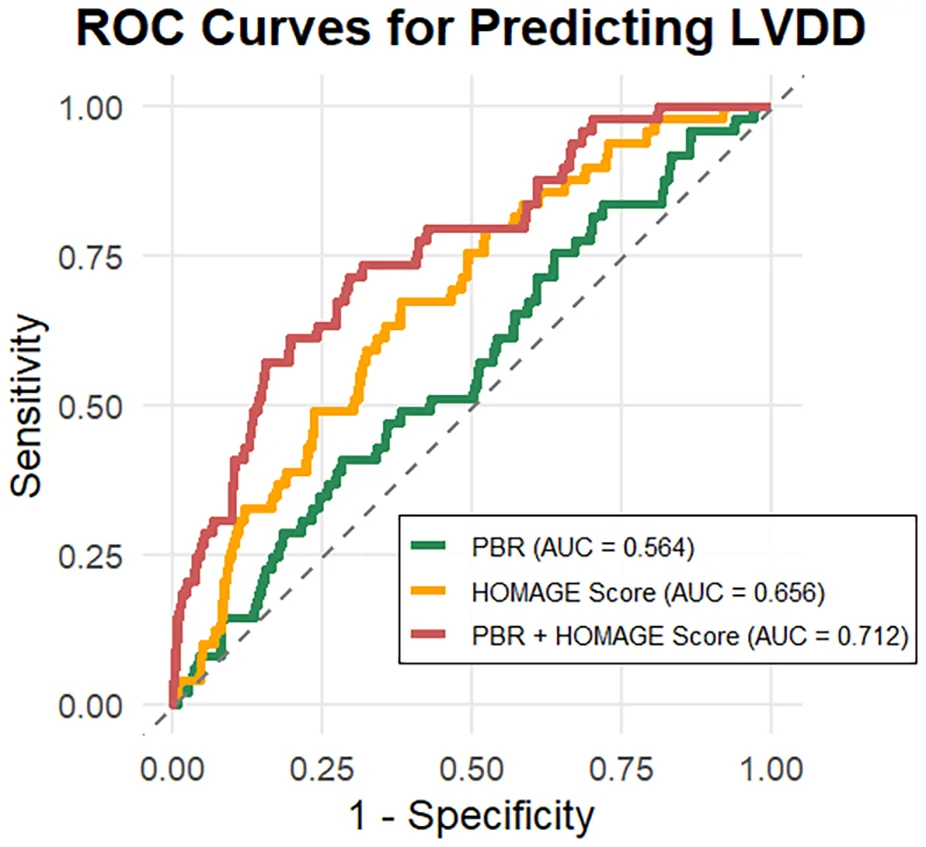
Receiver operating characteristic curves for discriminating left ventricular diastolic dysfunction. The blue line represents PBR alone (AUC 0.564, 95% CI 0.471-0.597; *P* < 0.01). The red line represents the HOMAGE score alone (AUC 0.656, 95% CI 0.576-0.735; *P* < 0.01). The green line represents the combined model (AUC 0.712, 95% CI 0.642-0.782; *P* < 0.01). DeLong's test confirmed a significant improvement in AUC when PBR was added to the HOMAGE score (*P* = 0.04). Optimal cut-off values were determined by the Youden index: 2.29 μm for PBR and 11.65 for the HOMAGE score, yielding sensitities/specificities of 63.5%/53.4% and 68.6%/59.6%, respectively. The combined model achieved a sensitivity of 70.5% and a specificity of 69.8%. AUC, area under the curve; CI, confidence interval; HOMAGE, Heart ‘OMics' in AGEing; LVDD, left ventricular diastolic dysfunction; PBR, perfused boundary region.

We further applied net reclassification improvement (NRI) and integrated discrimination improvement (IDI) analyses to determine whether adding PBR to the HOMAGE score enhanced discriminative capacity for LVDD. The results demonstrated that PBR significantly improved the discriminative value of the HOMAGE score alone (NRI: 0.73, SE: 0.32, 95% CI: 0.13-1.32, *P* = 0.02; IDI: 0.11, SE: 0.06, 95% CI: 0.01-0.22, *P* = 0.03).

### Relationship between EG barrier impairment and annual changes in diastolic function

Of the 503 participants enrolled at baseline, 460 (91.5%) completed the follow-up echocardiographic examination after a median observation period of 23 months (Figure 3, participant flow diagram). To address potential attrition bias, baseline characteristics were compared between participants who completed follow-up and those lost to follow-up. Baseline characteristics were well balanced between participants who completed follow-up and those who were lost to follow-up (43 participants, 8.5%), with no significant differences in age, sex, PBR, HOMAGE score, comorbidities, or baseline diastolic function grade (all *P* > 0.05; Table 4), suggesting that attrition bias was unlikely to substantially affect the longitudinal findings. This baseline comparison serves as a sensitivity analysis against informative missingness. Linear mixed models, adjusted for baseline age, sex, BMI, DM, and CKD, were used to estimate the annual rate of change in diastolic function parameters for the entire cohort, as well as for participants stratified into the HPG and LPG using a cut-off of 2.29 μm to maximize the discriminative separation between groups. The results are presented in Table 5.

**Figure 3:**
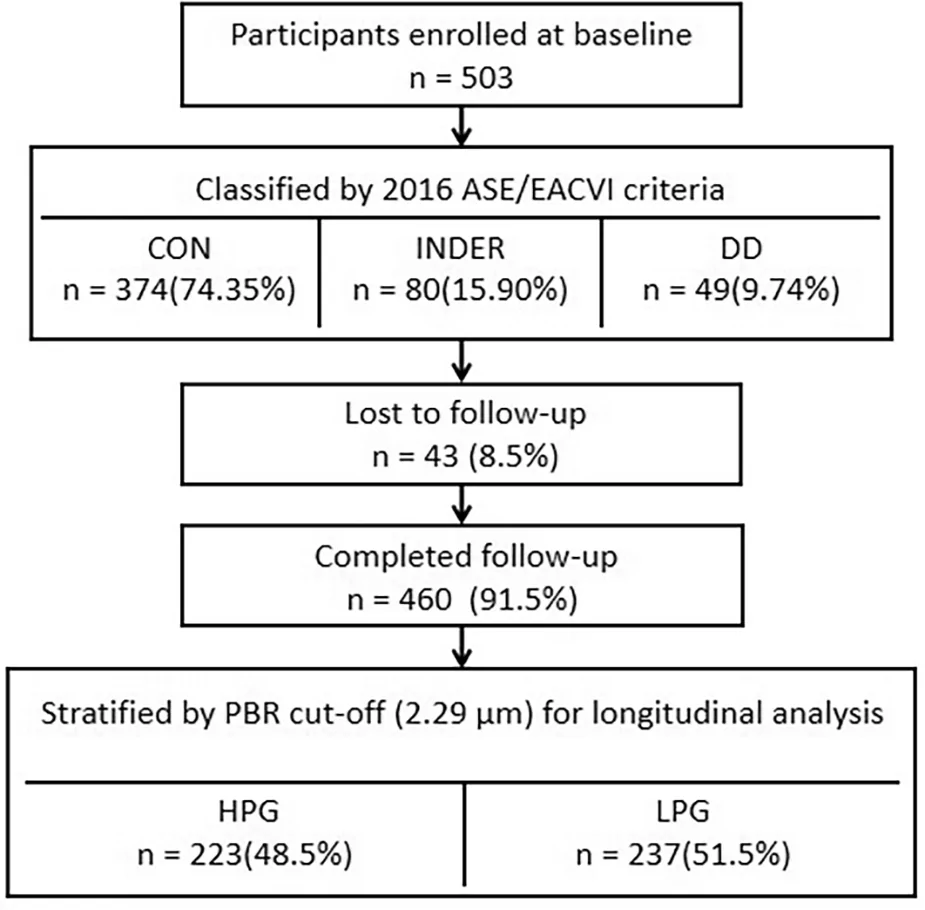
Participant flow diagram. Of 503 participants enrolled at baseline, 460 (91.5%) completed the follow-up echocardiographic examination after a median observation period of 23 months. For longitudinal analysis, participants were stratified into the high-PBR group (HPG, *n* = 223) and the low-PBR group (LPG, *n* = 237) using the ROC-derived optimal cut-off of 2.29 μm. ASE, American Society of Echocardiography; CON, normal diastolic function; DD, diastolic dysfunction; EACVI, European Association of Cardiovascular Imaging; HPG, high PBR group; INDER, indeterminate diastolic function; LPG, low PBR group; PBR, perfused boundary region.

**Table 4:** Baseline characteristics of participants who completed follow-up versus those lost to follow-up.

Characteristic	Completed follow-up (*n* = 460)	Lost to follow-up (*n* = 43)	*P* value
Demographics			
Age, years	65.9 ± 4.8	66.5 ± 5.2	0.45
Male, *n* (%)	374 (81.3)	36 (83.72)	0.48
Body mass index, kg/m^2^	26.5 (24.1–29.3)	26.2 (23.8–29.0)	0.60
Current smoker, *n* (%)	153 (33.26)	13 (30.23)	0.52
Comorbidities, *n* (%)			
Diabetes mellitus	72 (15.6)	8 (18.6)	0.55
Hypertension	190 (41.3)	18 (41.86)	0.84
Chronic kidney disease	44 (9.6)	5 (11.6)	0.65
Laboratory			
Fasting glucose, mmol/L	5.6 (5.1–6.4)	5.7 (5.2–6.5)	0.42
Total cholesterol, mmol/L	4.5 (3.9–5.2)	4.4 (3.8–5.1)	0.50
LDL cholesterol, mmol/L	2.7 (2.2–3.3)	2.6 (2.1–3.2)	0.38
HDL cholesterol, mmol/L	1.2 (1.0–1.4)	1.2 (1.0–1.4)	0.88
Triglycerides, mmol/L	1.5 (1.1–2.1)	1.6 (1.2–2.2)	0.45
Creatinine, μmol/L	71 (62–82)	72 (63–83)	0.40
eGFR, mL/min/1.73 m^2^	84 (72–96)	82 (70–94)	0.35
NT-proBNP, pg/mL	68 (53–98)	66 (52–93)	0.25
PBR measurement			
PBR, μm	2.07 (1.75–2.46)	2.10 (1.78–2.48)	0.50
Echocardiographic parameters			
LVEF, %	62 (58–66)	63 (59–67)	0.30
LV mass index, g/m^2^	84 (75–104)	85 (76–105)	0.55
LAVI, mL/m^2^	33 (30–39)	32 (29–38)	0.48
E/A ratio	0.96 (0.83–1.11)	0.95 (0.82–1.10)	0.60
Average E/e'	11.12 (10.15–12.23)	11.05 (10.08–12.18)	0.52
Septal e', cm/s	8.19 (7.11–9.20)	8.10 (7.02–9.15)	0.48
Lateral e', cm/s	9.81 (8.77–10.41)	9.75 (8.70–10.35)	0.50
TR velocity, m/s	2.55 (2.47–2.63)	2.56 (2.48–2.64)	0.44
Diastolic function, *n* (%)			
Normal (CON)	343 (74.6)	31 (72.1)	0.78
Indeterminate (INDER)	73 (15.9)	7 (16.3)	0.92
Diastolic dysfunction (DD)	44 (9.6)	5 (11.6)	0.65
HOMAGE score	9.88 (7.92–13.01)	9.82 (8.09–13.06)	0.94

Data are presented as mean ± SD, median (IQR), or *n* (%). *P* values were calculated using Student's t-test, Mann–Whitney U test, or *χ*^2^ test, as appropriate. No significant differences were observed between the two groups in any baseline characteristic (all *P* > 0.05), suggesting that attrition bias was unlikely to substantially affect the longitudinal findings. Abbreviations are as defined in Table 1.

**Table 5:** Annual changes in echocardiographic diastolic parameters according to baseline perfused boundary region level.

Echocardiographic parameter	All participants (*n* = 460)	HPG (*n* = 223)	LPG (*n* = 237)	*P* for trend*
Average E/e'	0.25 (0.07, 0.43)†	0.41 (0.17, 0.66)†	0.08 (−0.03, 0.31)	<0.01
Septal e' (cm/s)	−0.26 (−0.35, −0.17)†	−0.38 (−0.53, −0.24)†	−0.13 (−0.25, −0.02)†	<0.01
Lateral e' (cm/s)	−0.32 (−0.44, −0.21)†	−0.46 (−0.63, −0.29)†	−0.18 (−0.33, −0.02)†	<0.01
LAVI (mL/m^2^)	0.50 (0.12–0.88)	0.54 (0.01, 1.08)†	0.47 (−0.34, 0.79)	0.09
TR velocity (m/s)	0.02 (−0.01, 0.05)	0.02 (−0.01, 0.06)	0.03 (−0.01, 0.06)	0.14

Values are mean annual change (95% confidence interval), adjusted for baseline age, sex, BMI, diabetes mellitus, chronic kidney disease, and the baseline value of the respective echocardiographic parameter. *P for trend represents the *P* value for the time  ×  PBR-group interaction term in the linear mixed-effects model. †Significant within-group change (*P* < 0.05). BMI, body mass index; HPG, high PBR group; LAVI, left atrial volume index; LPG, low PBR group; PBR, perfused boundary region; TR, tricuspid regurgitation.

Over the follow-up period, patients in the HPG exhibited a significantly faster annual deterioration in the E/e' ratio, septal e', and lateral e' compared with those in the LPG (all P for trend < 0.01). Notably, although septal e' and lateral e' showed significant annual declines in both groups, the annual increase in E/e' was significant only in the HPG (0.41, 95% CI: 0.17-0.66), but not in the LPG (0.08, 95% CI: −0.03 to 0.31). In addition, LAVI showed a significant annual increase in the overall cohort and in the HPG, but not in the LPG. TR velocity showed no significant annual changes in any of the groups. No significant differences in yearly trends were detected between the HPG and LPG for either parameter (*P* for trend = 0.09 and 0.14, respectively).

## Discussion

This study provides novel evidence linking non-invasive assessment of EG integrity—quantified by PBR of sublingual microvessels—to both the presence and longitudinal progression of ALVDD in an elderly Chinese cohort without overt heart failure. Three principal findings emerged. First, higher PBR values, indicating reduced EG barrier integrity, were independently associated with a diagnosis of ALVDD after adjustment for traditional cardiovascular risk factors and the HOMAGE score. Second, adding PBR to the HOMAGE score significantly improved discrimination of ALVDD, as reflected by an enhanced area under the ROC curve, net reclassification improvement, and integrated discrimination improvement. Third, over a median follow-up of nearly two years, individuals with baseline PBR above the cohort median exhibited a significantly faster annual deterioration of key echocardiographic indices of diastolic function—specifically a rise in E/e' and a decline in septal and lateral e'—compared with those with lower PBR. Collectively, these observations position PBR as a potentially valuable predictor of evolving diastolic impairment and a possible intervention target.

DD is a crucial prognostic parameter for all-cause mortality and plays a key role in HF occurrence and prognosis, regardless of LVEF (19). Improvement in diastolic function is closely associated with cardiovascular outcomes (20, 21). A previous study indicated that 13% of patients experienced changes in diastolic function grade during follow-up (22). Therefore, exploring ALVDD-related risk factors is of great importance, especially since patients with ALVDD have a 70% higher risk of progressing to HFpEF than controls (3).

According to the 2016 ASE/EACVI criteria, the diagnostic threshold for DD is more stringent than that of the 2009 version, resulting in a substantially lower detection rate. In a community-based epidemiological survey of relatively healthy individuals aged over 45 years, Almeida and colleagues reported that the prevalence of ALVDD diagnosed according to the 2016 guidelines was only approximately 1.4% (23). By contrast, another investigation focusing on patients with diabetes mellitus found a higher ALVDD prevalence of 7.2% (24). In the present cohort, the observed prevalence of ALVDD was 9.74%. Given that advancing age is a well-established risk factor for DD development, the relatively elevated incidence in our study is likely attributable to the older mean age of the enrolled participants.

The absence of significant NT-proBNP elevation in ALVDD warrants comment. Our cohort comprised asymptomatic elderly with low NT-proBNP levels, which may lack sensitivity for early diastolic impairment. Additionally, our ALVDD group likely represents a very early stage, not yet associated with substantial natriuretic peptide elevation, and the small number of events limited statistical power to detect modest trends.

Although the single-centre design and inclusion of retired workers may limit generalisability, our cohort represents a clinically relevant high-risk population: urban elderly Chinese individuals with clustering of cardiometabolic risk factors. The ALVDD prevalence of 9.74% aligns with the 7%-10% range reported in other high-risk cohorts (23, 24), and subgroup analyses confirmed consistent associations across all strata. Nevertheless, external validation in geographically diverse populations is required before clinical translation, particularly given the exploratory nature of the PBR cut-off (2.29μm).

Currently, there is no universally accepted reference range for PBR measured by sublingual sidestream dark field imaging. A study conducted in a physically healthy cohort reported a median PBR of approximately 1.89μm (IQR: 1.71-2.06 μm) (25), whereas the median PBR in our elderly, multimorbid population was 2.08μm (IQR: 1.76-2.47μm). This elevation is consistent with the well established effects of aging, diabetes, and chronic comorbidities on glycocalyx degradation (9, 26, 27), and further supports the notion that PBR reflects cumulative microvascular injury rather than a fixed physiological constant. Notably, Brands et al. reported that coronary artery disease was associated with impaired sublingual glycocalyx barrier function predominantly in women (13). Our observation of higher PBR in females is directionally consistent with this finding, though our asymptomatic elderly cohort without overt CAD may represent an earlier stage of microvascular injury, potentially influenced by menopausal status and cumulative risk factor burden.

The optimal cut-off value of 2.29μm derived from our ROC analysis should be interpreted with caution. This threshold is exploratory and study specific. It was optimised to discriminate ALVDD in our particular cohort and may not generalise to other populations with different age structures, comorbidity profiles, or ethnic backgrounds. Therefore, we emphasise that this cut-off requires external validation in independent, prospective cohorts before it can be proposed for clinical risk stratification. Similarly, we refrain from defining a ‘normal' versus ‘abnormal' PBR range based on our data alone. Instead, we have used the continuous PBR variable as the primary exposure in regression models to capture the dose response relationship, and used dichotomisation (median based or ROC derived) solely for descriptive and hypothesis generating purposes. Establishing normative PBR reference values across age, sex, and comorbidity strata, along with standardisation of measurement protocols, should be a priority for future multicentre studies to facilitate clinical translation.

ALVDD serves as a precursor to HF, particularly HFpEF, which is highly prevalent in the elderly population. Although the relationship between PBR and the subsequent development of HFpEF has not been extensively studied, emerging evidence suggests that glycocalyx disintegration—quantified by an enlarged PBR—is closely linked to adverse outcomes in patients with established HF. A recent prospective study by Wadowski et al. demonstrated that patients with chronic heart failure and elevated PBR values exhibited significantly worse two-year overall survival compared with those with preserved glycocalyx integrity (28). Moreover, higher PBR was identified as an independent predictor of all-cause mortality in chronic heart failure, underscoring its prognostic relevance (29). Other markers reflecting the condition of the glycocalyx have been confirmed to be associated with diastolic function (30). The findings of the present study provide a mechanistic explanation for these observations from the perspective of diastolic function: elevated PBR, reflecting reduced EG barrier function, is independently associated with worse diastolic parameters. This association suggests that glycocalyx damage may serve as an early link between microvascular endothelial dysfunction and subsequent diastolic deterioration, thereby offering a potential pathway through which PBR contributes to poor prognosis in patients with HFpEF. Previous studies have linked PBR to adverse outcomes in established HF (28, 29), but largely unexplored the asymptomatic stage. Our study extends this evidence by focusing on ALVDD and providing longitudinal data on functional deterioration.

With regard to the underlying mechanisms, we hypothesize that an elevated PBR—indicative of glycocalyx thinning or disruption—compromises the protective barrier function of the microvascular endothelium, thereby exacerbating endothelial vulnerability to inflammatory insults. EG degradation has been identified as an early pathophysiological event in HFpEF, triggered by comorbidities such as chronic low-grade inflammation, oxidative stress, and metabolic derangements (9). EG degradation is mainly driven by matrix metalloproteinases, specific degradation enzymes (heparanase, hyaluronidase), and reactive oxygen species (7, 8). Disintegration of the glycocalyx leads to increased vascular permeability, enhanced leukocyte adhesion, and reduced nitric oxide bioavailability at the endothelial level. Through the NO-sGC-cGMP-PKG signaling cascade, diminished nitric oxide availability reduces PKG activity in adjacent cardiomyocytes, which in turn promotes cardiomyocyte hypertrophy, increases myocyte stiffness due to reduced PKG-mediated phosphorylation of titin, and facilitates interstitial collagen deposition via activation of profibrotic mediators such as TGF-*β* and the PDGF-PDGFR axis (7). The resulting impairment of active relaxation and passive compliance ultimately manifests as diastolic dysfunction. Emerging evidence from large cohort studies substantiates these mechanistic links at the biomarker level. Lagrange et al. recently demonstrated that higher circulating levels of glycocalyx degradation biomarkers, particularly perlecan and thrombomodulin, are associated with features of diastolic dysfunction in both population-based and HFpEF cohorts, and predict poor outcomes in patients with established HFpEF (30). Similarly, elevated syndecan-1 and hyaluronan levels have been shown to correlate with adverse cardiac remodelling and increased cardiovascular event rates in HFpEF (31). These findings suggest that glycocalyx degradation may represent an early step in the pathological continuum from microvascular endothelial dysfunction to diastolic impairment and overt HFpEF (27, 29). Collectively, these findings support the concept that elevated PBR may serve as a non-invasive indicator of systemic microvascular endothelial dysfunction and may be involved in the pathophysiology of HFpEF through the NO-cGMP-PKG pathway, consistent with the Paulus-Tschöpe paradigm that positions coronary microvascular endothelial inflammation as a central driver of myocardial remodelling, although causal inference cannot be established from observational data (7).

The longitudinal component of this study is particularly noteworthy. The finding that participants with higher PBR experienced more pronounced worsening of E/e' and e' velocities over two years provides temporal evidence that EG damage precedes or accompanies functional deterioration. This aligns with the hypothesis that microvascular endothelial inflammation acts as a driver of progressive myocardial stiffening, a central mechanism in the development of heart failure with preserved ejection fraction. The absence of significant changes in LAVI or TR velocity during the same follow-up period may indicate that these parameters require a longer observation time to manifest detectable alterations, or that they are less sensitive to short-term EG changes.

From a clinical perspective, the moderate effect size and modest AUC improvement should be interpreted in the context of the intended use case. PBR is not proposed as a standalone diagnostic test for ALVDD, nor as a replacement for established risk scores. Rather, its primary value lies in risk reclassification among individuals with intermediate HOMAGE scores, as reflected by the significant NRI of 0.73, meaning that approximately 73% of participants were correctly reclassified when PBR was added to the model. This suggests that PBR may be most useful in situations where traditional risk factors leave clinical uncertainty, such as in community-based screening or outpatient geriatric assessments.

This finding also suggests that EG integrity may play a role in the pathophysiology of ALVDD and that strategies aimed at preserving the EG warrant further investigation as a potential approach to mitigate the risk of ALVDD and subsequent HF. Emerging evidence indicates that the EG is a dynamic and potentially modifiable target. Glycocalyx-protective interventions, including dietary precursor supplementation, sulodexide, SGLT2 inhibitors, and GLP-1 receptor agonists, have been shown to reduce PBR in various populations (32–35). These findings raise the hypothesis that PBR could serve as a treatment target, though this requires confirmation in prospective interventional trials.

Several limitations should be acknowledged. First, although the single centre design and inclusion of retired workers limit generalizability, our cohort represents a clinically relevant high-risk population, with subgroup analyses confirming consistent associations across strata. External validation in diverse populations is required. Second, we did not directly measure coronary microvascular function (e.g., by invasive or angiographic indices), and the association between PBR and coronary microcirculation is inferred from previous validation studies rather than directly assessed in this cohort. Third, the optimal PBR cut-off of 2.29μm derived from ROC analysis was exploratory and derived from a single cohort. It requires external validation in independent, prospectively recruited populations before it can be considered for clinical application. The cutoff should therefore be interpreted as hypothesis generating rather than definitive. Fourth, despite adjustment for multiple confounders, residual confounding—particularly from unmeasured inflammatory biomarkers such as high-sensitivity C-reactive protein or interleukin-6—cannot be excluded. Low-grade systemic inflammation is common in the elderly and may simultaneously promote EG degradation and diastolic dysfunction. Future studies should incorporate such biomarkers to explore mediating pathways. Fifth, we did not repeat PBR or inflammatory biomarker measurements at follow-up. Consequently, we cannot determine whether changes in glycocalyx integrity over time directly parallel or precede the observed diastolic deterioration.

## Conclusion

In conclusion, this study demonstrates that EG barrier impairment, as quantified by sublingual PBR, is independently associated with both the current presence and two-year progression of ALVDD in an elderly Chinese population. PBR may serve as a complementary marker for risk reclassification, particularly in intermediate-risk individuals, and glycocalyx-protective interventions warrant further investigation as a potential therapeutic strategy.

## Data Availability

The raw data supporting the conclusions of this article will be made available by the authors, without undue reservation.
