# Supplementary material for: Sublingual microcirculation-derived glycocalyx barrier impairment independently predicts progression of asymptomatic left ventricular diastolic dysfunction in elderly Chinese adults
Source: Front Cardiovasc Med. 2026 Sep 24;13:1933360. doi: 10.3389/fcvm.2026.1933360 (PMC13645779; doi:10.3389/fcvm.2026.1933360)
Supplement: Supplementary file 1 [file Table1.doc]

## **Supplementary Table S1. Subgroup analyses for the association between perfused boundary region and asymptomatic left ventricular diastolic dysfunction**

| Subgroup | DD / Total, n | OR (95% CI), per 0.1μm PBR increase | P for interaction |
| --- | --- | --- | --- |
| **Sex** |  |  | **0.41** |
| Male | 32 / 410 | 1.11 (1.05-1.17) |  |
| Female | 17 / 93 | 1.14 (1.05-1.23) |  |
| **Age** |  |  | **0.56** |
| <65 years | 18 / 243 | 1.12 (1.04-1.20) |  |
| ≥65 years | 31 / 260 | 1.11 (1.05-1.18) |  |
| **Diabetes mellitus** |  |  | **0.47** |
| Yes | 10 / 80 | 1.10 (1.01-1.19) |  |
| No | 39 / 423 | 1.12 (1.06-1.19) |  |
| **Hypertension** |  |  | **0.73** |
| Yes | 23 / 215 | 1.11 (1.04-1.18) |  |
| No | 26 / 288 | 1.13 (1.06-1.20) |  |
| **Smoking status** |  |  | **0.52** |
| Current smoker | 16 / 166 | 1.10 (1.02–1.19) |  |
| Non-smoker | 33 / 337 | 1.12 (1.06–1.19) |  |

All ORs were derived from multivariable logistic regression models adjusted for the HOMAGE score (which integrates age, sex, BMI, smoking status, diabetes, antihypertensive medication use, systolic blood pressure, heart rate, and creatinine), except that the stratification variable itself was omitted from the respective subgroup model to avoid collinearity. For example, in the sex‑stratified models, sex was not included as a covariate; in the age‑stratified models, age was excluded, and so forth. The "P for interaction" was calculated by introducing a multiplicative interaction term (PBR × subgroup variable) into the fully adjusted model. A two‑sided P value <0.10 for interaction was considered suggestive of effect modification, given the limited sample size and number of events; none of the interaction tests reached this threshold, indicating no significant heterogeneity across strata. The subgroup‑specific event counts are provided to allow readers to assess the stability of the estimates despite the modest overall event number (n = 49). Abbreviations: ALVDD, asymptomatic left ventricular diastolic dysfunction; PBR, perfused boundary region; OR, odds ratio; CI, confidence interval.
